# Reporting of HIV-infected pregnant women: estimates from a Brazilian study

**DOI:** 10.11606/S1518-8787.2018052017439

**Published:** 2018-04-04

**Authors:** Rosa Maria Soares Madeira Domingues, Valéria Saraceni, Maria do Carmo Leal

**Affiliations:** I Fundação Oswaldo Cruz. Instituto Nacional de Infectologia Evandro Chagas. Laboratório de Pesquisa Clínica em DST/Aids. Rio de Janeiro, RJ, Brasil; II Secretaria Municipal de Saúde do Rio de Janeiro. Superintendência de Vigilância em Saúde. Coordenação de Análise da Situação de Saúde. Rio de Janeiro, RJ, Brasil; III Fundação Oswaldo Cruz. Escola Nacional de Saúde Pública Sérgio Arouca. Departamento de Epidemiologia e Métodos Quantitativos em Saúde. Rio de Janeiro, RJ, Brasil

**Keywords:** Pregnant Women, HIV Infections, diagnosis, Prenatal Care, Health Information Systems, Gestantes, Infecções por HIV, diagnóstico, Cuidado Pré-Natal, Sistemas de Informação em Saúde, Serviços de Vigilância Epidemiológica

## Abstract

**OBJECTIVE:**

To estimate the coverage of the reporting of cases of HIV-infected pregnant women, to estimate the increase in the coverage of the reporting with the routine search of data in other Brazilian health information systems, and to identify missed opportunities for identification of HIV-infected pregnant women in Brazilian maternity hospitals.

**METHODS:**

This is a descriptive study on the linkage of Brazilian databases with primary data from the “*Nascer no Brasil*” study and secondary database collection from national health information systems. The “*Nascer no Brasil*” is a national-based study carried out in 2011–2012 with 23,894 pregnant women, which identified HIV-infected pregnant women using prenatal and medical records. We searched for cases of HIV-infected pregnant women identified in the “*Nascer no Brasil*” study in the Information System of Notifiable Diseases, the Control System for Laboratory Tests of the National CD4+/CD8+ Lymphocyte Count and HIV Viral Load Network, and the Logistics Control System for Medications. We used the OpenRecLink software for the linkage of databases. We estimated the notification coverage, with the respective confidence interval, of the evaluated Brazilian health information systems.

**RESULTS:**

We estimated the coverage of the reporting of HIV-infected pregnant women in the Information System of Notifiable Diseases as 57.1% (95%CI 42.9–70.2), and we located 89.3% of the HIV-infected pregnant women (95%CI 81.2–94.2) in some of the Brazilian health information systems researched. The search in other national health information systems would result in an increase of 57.1% of the reported cases. We identified no missed opportunities for the diagnosis of HIV+ in pregnant women in the maternity hospitals evaluated by the “*Nascer no Brasil*” study.

**CONCLUSIONS:**

The routine search for information in other Brazilian health information systems, a procedure carried out by the Ministry of Health for cases of AIDS in adults and children, should be adopted for cases of HIV in pregnancy.

## RESUMO

**OBJETIVO:**

Estimar a cobertura de notificação de casos de infecção pelo HIV em gestantes, o aumento na cobertura de notificação a ser obtido pela busca rotineira de dados em outros sistemas de informação nacionais, e identificar oportunidades perdidas de identificação de gestantes HIV+ em maternidades brasileiras.

**MÉTODOS:**

Estudo descritivo, de relacionamento de base de dados de estudo nacional com coleta primária de dados (estudo “Nascer no Brasil”), e de base de dados secundários de sistemas de informação nacionais. O estudo “Nascer no Brasil” é um estudo de base nacional, realizado em 2011–2012, com 23.894 puérperas, que identificou gestantes infectadas pelo HIV, utilizando registros em cartão de pré-natal e prontuário. Casos de gestantes com infecção pelo HIV identificadas no estudo “Nascer no Brasil” foram pesquisadas no Sistema de Informação de Agravos de Notificação, Sistema de Controle de Exames Laboratoriais da Rede Nacional de Contagem de Linfócitos CD4+/CD8+ e Carga Viral, e Sistema de Controle Logístico de Medicamentos. Utilizou-se o *software* OpenRecLink para relacionamento das bases de dados. Foi estimada a cobertura de notificação, com respectivo intervalo de confiança, dos sistemas de informação nacionais avaliados.

**RESULTADOS:**

A cobertura de notificação de HIV em gestantes no Sistema de Informação de Agravos de Notificação foi estimada em 57,1% (IC95% 42,9–70,2), e 89,3% das gestantes HIV+ (IC95% 81,2–94,2) foram localizadas em algum dos sistemas de informação nacionais pesquisados. A busca em outros sistemas de informação nacionais resultaria em aumento de 57,1% dos casos notificados. Não foram identificadas oportunidades perdidas de diagnóstico de gestantes HIV+ nas maternidades avaliadas pelo estudo “Nascer no Brasil”.

**CONCLUSÕES:**

A busca rotineira de informação em outros sistemas de informação nacionais, procedimento utilizado pelo Ministério da Saúde para casos de Aids em adultos e em crianças, deve ser adotado para os casos de infecção pelo HIV na gestação.

## INTRODUCTION

The HIV infection in pregnancy is a disease of compulsory reporting in Brazil since 2006. The rate of detection of cases of HIV-infected pregnant women in the country has increased in the last ten years, reaching 2.7 per 1,000 live births in 2015. We can observe differences in detection rates in different regions. The North and Northeast regions of Brazil showed the highest increase in this rate from 2006 to 2015, while the South region had the highest rate of detection in the entire period, with a value 2.2 times greater than for the country in 2015^1^.

Despite improvements in the reporting of such cases, underreporting is still estimated to be high. Sentinel studies on pregnant women carried out in 2006^2^ and 2010/2012^3^ in public hospitals estimated a prevalence of HIV-infected pregnant women in the country at 0.41% and 0.38%, respectively. Considering an estimated 11,070 cases per year (calculated from the prevalence of 0.38% of HIV-infected pregnant women, measured in 2010/2012) and the notification of 7,901 cases in 2015, we can estimate that approximately 70.0% of the cases of HIV-infected pregnant women were reported. This indicates difficulties in the diagnosis or reporting of cases.

Data from four information systems – Information System of Notifiable Diseases (SINAN), Mortality Information System (SIM), Control System for Laboratory Tests of the National CD4+/CD8+ Lymphocyte Count and HIV Viral Load Network (SISCEL), and Logistic System for Medications (SICLOM) – are routinely used as a source for data collection by the Ministry of Health to increase and qualify the data on the reporting of AIDS in children and adults. Considering the historical series from 2000 to 2016, out of a total of 634,051 cases of registered AIDS, only 70.3% came from the SINAN, which highlights the importance of this strategy to better estimate cases. However, this routine is not implemented for cases of HIV infection in pregnant women.

The “*Nascer no Brasil*”^4^ is a national hospital-based study carried out in 2011–2012, which allowed the identification of HIV-infected pregnant women from data recorded on the prenatal and hospital records. Considering the flaws in the system for the reporting of cases of HIV-infected pregnant women, this study aimed to estimate the coverage of the reporting of cases of HIV-infected pregnant women in the SINAN, to estimate the increase in the coverage of the reporting obtained by the routine search of data in other information systems (SIM, SISCEL, SICLOM), and to identify missed opportunities to prevent vertical transmission in Brazilian maternity hospitals using as reference the results of the “*Nascer no Brasil*” study.

## METHODS

This is a descriptive study of the linkage of databases with primary data from the “*Nascer no Brasil*” study and secondary database collection from Brazilian Health Information Systems (SINAN, SIM, SISCEL, SICLOM).

The “*Nascer no Brasil*” study is a national hospital-based study, carried out between February 2011 and October 2012. It aimed to evaluate the prenatal, delivery, and newborn care and the results of this care. The sample of the study was 23,894 women hospitalized in 266 public and private hospitals in all regions of Brazil. All women who had live births in the hospital with a fetus that had any gestational age or weight or who had a stillbirth in which the fetus was above the gestational age of 22 weeks or weighed more than 500 grams were considered as eligible for the study. More information about the study design and sample calculation can be obtained in Leal^4^ and Vasconcellos^5^.

As a secondary database, we used the Brazilian Health Information Systems: SINAN, SISCEL, and SICLOM. We also planned to use the SIM database, but we could not obtain it.

Cases of “HIV-infected pregnant women,” “HIV-exposed children”, and “AIDS” are of compulsory reporting in Brazil. Specific models of report forms for each of these diseases are used nationally by the SINAN. The reported cases are processed by the Municipal and State Health Departments, and the data are sent to the Ministry of Health, which provides the national data regarding the year of diagnosis of the disease.

The SICLOM has the goal of managing the logistics of the antiretroviral therapy (ART) in Brazil. All antiretroviral therapy provided to HIV-infected pregnant women, HIV-exposed children, and persons (adults and children) with AIDS are registered in this system.

The Control System for Laboratory Tests of CD4/CD8 and Viral Load has been used in the country since 1997 to control the viral load and CD4/CD8 counts in persons living with HIV and AIDS. The SISCEL is used in all Brazilian states and all data are stored in the central database, which is in the Department of STD, AIDS, and Viral Hepatitis.

For the first objective, “to estimate the coverage of the reporting of cases of HIV-infected pregnant women”, we carried out a nominal search of the cases of pregnant women diagnosed with HIV infection identified in the “*Nascer no Brasil*” study and in the SINAN.

In order to identify the cases of HIV-infected pregnant women in the “*Nascer no Brasil*” study, we used the data on the prenatal and hospital records according to the following criteria^6^: a) the results of the HIV serological reagents present on the prenatal record (two rapid tests or ELISA + immunofluorescence or ELISA + Western Blot), or b) records in the hospital medical files of any of the following situations: diagnosis of HIV infection, indication of cesarean section because of HIV infection, use of AZT during labor or delivery, use of AZT syrup by the newborn, cessation of breastfeeding because of maternal HIV infection, registration of the diagnosis of “child exposed to HIV”.

To identify HIV-infected pregnant women in the SINAN, we selected the cases of “HIV in gestation” reported in the SINAN from May 1, 2010 to October 31, 2012. Data collection from the “*Nascer no Brasil*” study occurred from February/2011 to October/2012. The inclusion of 2010 aimed at identifying pregnant women with delivery after February 2011 and who were reported in the SINAN during pregnancy in 2010.

For the second objective, “to estimate the increase in the coverage of the reporting with the routine search of data in other health information systems”, we used data from the SINAN, SICLOM, and SISCEL systems. In the SINAN, we researched cases of AIDS in adults. This research aimed to identify women previously reported with AIDS who had not been notified as HIV-infected pregnant women during gestation. For this search, we used the data of the SINAN until October 31, 2012, which is the last date for data collection of the “*Nascer no Brasil*” study. We planned to search for cases of HIV-exposed children to identify children exposed during gestation and delivery, whose mother had not been reported. However, this survey record is not implemented and there is no database available for consultation.

All cases recorded in the SICLOM or SISCEL of HIV-infected pregnant women, HIV-exposed children, or HIV/AIDS-infected children were selected in this study because they are specific systems for HIV-infected individuals. For HIV-infected pregnant women, we considered the records from May 1, 2010 to October 31, 2012 in order to identify pregnant women with delivery in 2011 and who may have been registered in the SISCEL or SICLOM during pregnancy in 2010. For HIV-exposed children, we considered the records from February 1, 2011 to December 31, 2012 in order to identify children born in October 2012 with antiretroviral therapy for six weeks after delivery. For the HIV-infected children, we used the period of February 1, 2011 to June 31, 2016.

We compared the cases identified in the systems to cases reported as HIV in pregnancy in the SINAN identified in the first objective.

For the third objective, “to identify missed opportunities to prevent vertical transmission in Brazilian maternity hospitals”, all pregnant women without diagnosis of HIV infection in the “*Nascer no Brasil*” study were related to the SINAN, SISCEL, and SICLOM systems. Cases of HIV infection in pregnancy and AIDS in adults reported in the SINAN and HIV-infected pregnant women registered in the SISCEL and SICLOM were considered as unidentified by maternity hospitals, representing missed opportunities to prevent the vertical transmission during childbirth care. We used the same search periods in each information system described in the second objective for the third objective.

We used the following procedures for the linkage of databases: 1) Cleaning and standardization of the variables present in the databases of the “*Nascer no Brasil*” study, SINAN AIDS adult previously related to the SIM, SINAN HIV-positive pregnant women, records in the SICLOM and SISCEL using Stata 11.0, and 2) Linkage of databases using OpenRecLink (ORL) software. Order of the linkage between databases: database *Nascer* and records in the SICLOM and SISCEL, database *Nascer* and SINAN HIV+ Pregnant Women, database *Nascer* and SINAN AIDS Adult.

The following procedures were used in all linkages:

- Variables for linkage: name of the pregnant woman, name of the mother, date of birth, city of residence (for visual inspection).
- Blocking:
  Step 1: FBLOCK (1st name) + LBLOCK (last name) + YEARBIR (year of birth)
  Step 2: FBLOCK + YEARBIR + FBLOCK_mother (1st name of the mother)
  Step 3: FBLOCK + LBLOCK + FBLOCK_mother
- Parameters of the variables for linkage:
  Name: Type “Approximate”, Correct “92”, Incorrect “1”, and Threshold “85”
  Name of the mother: Type “Approximate”, Correct “92”, Incorrect “1”, and Threshold “85”
  Date of birth: Type “Character”, Correct “90”, Incorrect “5”, and Threshold “65”, according to the RecLink III Manual^7^.

The addresses of the women found underwent visual inspection as conference. Given the small number of pairs found, the women not located were searched by name in the SINAN and SICLOM/SISCEL databases. After linkage, a final base was built that included the cases found in the various systems and the women included in the “*Nascer no Brasil*” study.

To calculate the coverage of the reporting of HIV-infected pregnant women, we estimated the proportion of pregnant women with diagnosis of HIV infection identified by the “*Nascer no Brasil*” study, reported in the SINAN in the country and by macroregion, with 95% confidence interval.

We described the cases identified in each Health Information System (HIS) to evaluate the increase in the coverage of the reporting that would be obtained with the routine use of other sources of information in addition to the SINAN (SISCEL, SICLOM, and SIM). In addition, we estimated the increase in the coverage that would be obtained with the routine use of these sources of information.

We verified the proportion of HIV-infected pregnant women (identified by the “*Nascer no Brasil*” study and the HIS) who were not identified by the maternity hospitals that participated in the “*Nascer no Brasil*” study to calculate the missed opportunities for the prevention of vertical transmission.

The “*Nascer no Brasil*” study used a complex sample design that was considered in all statistical analyses with the use of weighting and calibration procedures^6^ for all the data (numbers and proportions) present in this study. The analyses were performed with the statistical software IBM SPSS Statistics for Windows, version 19.0 (IBM Corp., Armonk, NY, USA).

This study has been approved by the Research Ethics Committee of the Escola Nacional de Saúde Pública Sérgio Arouca of the Fundação Oswaldo Cruz (Protocol 1.647.494/2016). The nominal bases of the described systems were provided by the Ministry of Health with the signing of a term of responsibility. We did not use the informed consent because this was a database analysis. Only the team members had access to the nominal databases provided by the Ministry of Health and we took every precaution to ensure the confidentiality of the information.

## RESULTS

**Figure 1:**
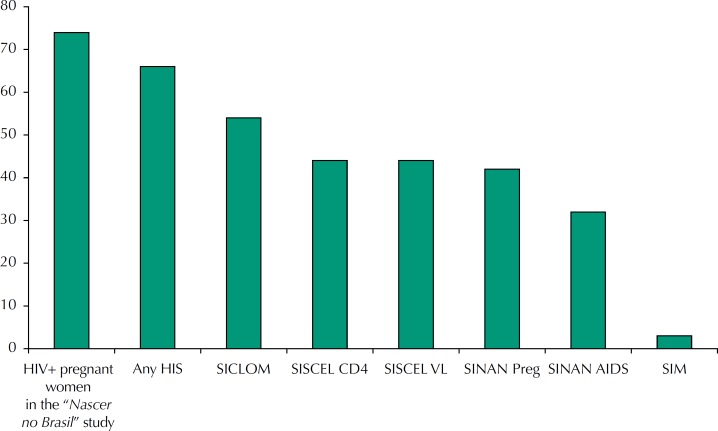
Number of HIV-infected pregnant women identified in the “Nascer no Brasil” study and in Brazilian Health Information Systems. Brazil, 2011 to 2012. HIS: Health Information System; SINAN Preg: Information System of Notifiable Diseases for HIV+ Pregnant women; SINAN AIDS: Information System of Notifiable Diseases for AIDS; SIM: Mortality Information System; SISCEL CD4: Control System for Laboratory Tests of the National CD4+/CD8+ Lymphocyte Count Network; SISCEL VL Control System for Laboratory Tests of the National HIV Viral Load Network; SICLOM: Logistics Control System for Medications

Of the total number of cases of HIV-infected pregnant women reported in the “*Nascer no Brasil*” study (n = 74), 89.3% were identified in one health information system: 73.1% in the SICLOM, 60.4% in the SISCEL Viral Load, 59.3% in the SISCEL CD4, 57.1% in the SINAN HIV Pregnant Women, 42.6% in the SINAN AIDS, and 3.7% in the SIM (Table 1, Figure 1). Most women were identified in more than one system, with a small proportion being recognized in only one information system: 3.9% in the SICLOM, 5.3% in the SINAN HIV Pregnant Women, and 1.1% in the SINAN AIDS.

**Table 1:** Proportion of cases of HIV-infected pregnant women identified in the “Nascer no Brasil” study, located in the SINAN, SICLOM, and SISCEL information systems. Brazil, 2011 to 2012.

Health Information System (HIS)	%	95%CI
SICLOM	73.1	61.9–82.0
SISCEL Viral Load	60.4	48.1–71.6
SISCEL CD4	59.3	46.9–70.6
SINAN HIV+ Pregnant Women	57.1	42.9–70.2
SINAN AIDS	42.6	31.6–54.5
SIM	3.7	1.0–12.0
Any Health Information System	89.3	81.2–94.2

SINAN: Information System of Notifiable Diseases; SISCEL: Control System for Laboratory Tests of the National CD4+/CD8+ Lymphocyte Count and HIV Viral Load Network; SICLOM: Logistics Control System for Medications; SIM: Mortality Information System

The identification of cases of HIV-infected pregnant women in some system ranged from 75.3% in the North region to 96.7% in the South region. We observed larger regional variations for the reporting of HIV-infected pregnant women in the SINAN. We observed the lowest coverage in the North region (20.2%) and the highest coverage in the Northeast region (79.5%) (Table 2).

**Table 2:** Proportion of HIV-infected pregnant women identified in the “Nascer no Brasil” study, according to location in one of the Brazilian Health Information Systems (SINAN AIDS, SICLOM, SISCEL) and in the SINAN “HIV+ Pregnant Women” system by Brazilian macroregion. Brazil, 2011 to 2012.

Region	Any Information System		SINAN HIV+ Pregnant Women	
	%	95%CI	%	95%CI
North	75.3	62.7–84.7	20.2	2.3–73.1
Northeast	84.5	79.6–88.4	79.5	73.8–84.3
Midwest	76.3	66.6–83.9	76.3	66.6–83.9
Southeast	92.1	55.9–99.1	54.9	25.4–81.3
South	96.7	77.1–99.6	57.0	42.0–70.8
Total	89.3	81.2–94.2	57.1	42.9–70.2

SINAN HIV+ Pregnant Women: Information System of Notifiable Diseases for HIV+ pregnant women; SICLOM: Logistics Control System for Medications; SISCEL: Control System for Laboratory Tests of the National CD4+/ CD8+ Lymphocyte Count and HIV Viral Load Network

**Figure 2:**
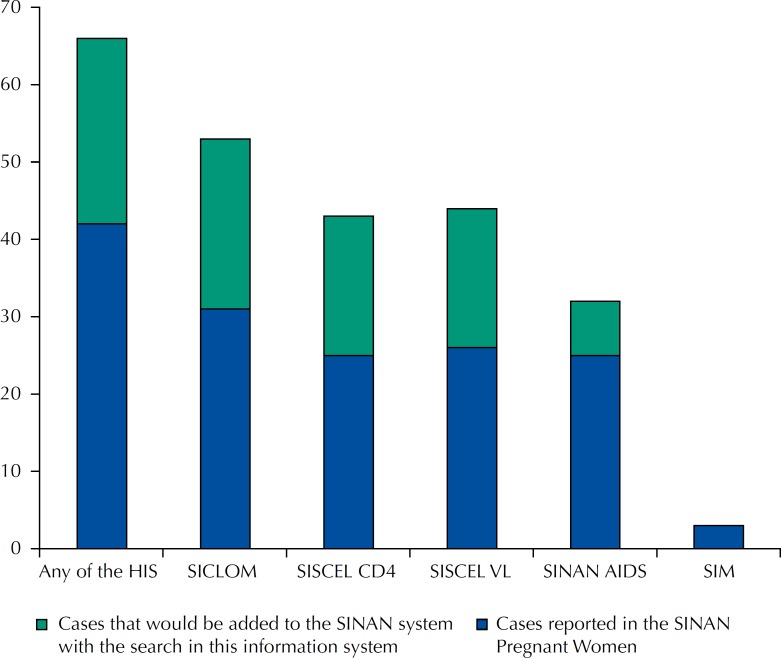
Cases reported in the SINAN for HIV in pregnancy and cases that would be added with the search in other Brazilian Health Information System. Brazil, 2011 to 2012. HIS: Health Information System; SINAN AIDS: Information System of Notifiable Diseases for AIDS; SIM: Mortality Information System; SISCEL CD4: Control System for Laboratory Tests of the National CD4+/CD8+ Lymphocyte Count Network; SISCEL VL Control System for Laboratory Tests of the National HIV Viral Load Network; SICLOM: Logistics Control System for Medications

Considering the cases identified in any of the Health Information Systems, the prevalence of HIV infection in pregnancy was estimated at 0.3% (95%CI 0.21–0.36). Using only the cases reported in the SINAN HIV Pregnant Women, the estimated prevalence of HIV infection in pregnancy was 0.2% (95%CI 0.13–0.23). With the routine search of cases in other information systems, the number of cases in the SINAN HIV Pregnant Women could increase to 57.1%. The largest increase was obtained with the SICLOM, which represented an increase of 52.4% in reported cases, followed by SISCEL CD4 and VL (42.9%), and SINAN AIDS (16.7%) (Table 3, Figure 2).

**Table 3:** Proportion of HIV+ pregnant women identified in the “Nascer no Brasil” study, according to notification in the SINAN “HIV+ Pregnant Women” system and identification in other Health Information Systems. Brazil, 2011 to 2012.

Health Information System (HIS)	SINAN HIV+ Pregnant Women		Total
	No (32)	Yes (42)	
	n (%)	n (%)	
SICLOM	22 (70.9)	31 (74.7)	53
SISCEL VL	18 (57.4)	26 (62.7)	44
SISCEL CD4	18 (57.4)	25 (60.7)	43
SINAN AIDS	7 (21.7)	25 (58.4)	32
SIM	0	3 (100)	3
Any HIS	24 (75.1)	42 (100)	66

SINAN: Information System of Notifiable Diseases; SIM: Mortality Information System; SISCEL: Control System for Laboratory Tests of the National CD4+/CD8+ Lymphocyte Count and HIV Viral Load Network; SICLOM: Logistics Control System for Medications

Of the cases of HIV-infected pregnant women identified in the Health Information Systems, six were not diagnosed by the maternity hospitals that participated in the “*Nascer no Brasil*” study. Of the six cases identified, five presented a diagnosis of HIV infection in a gestation after that evaluated by the “*Nascer no Brasil*” study, and one pregnant woman had the diagnosis in a previous pregnancy, presenting “non-reactive” serology for HIV in the current gestation, which represents probable false-positive diagnosis in the previous pregnancy.

## DISCUSSION

The result obtained, that is the reporting of 57.1% of the pregnant women in the “*Nascer no Brasil*” study in the SINAN HIV in pregnancy, is slightly lower than the estimated coverage of the reporting calculated from data on prevalence of sentinel studies with pregnant women (approximately 70.0%). From the small number of cases identified, the estimate obtained is not precise, with a wide confidence interval and, therefore, it should be analyzed with caution, especially for the macroregions of the country.

Previous studies have evaluated the reporting of cases of HIV-infected pregnant women in Brazil. Lemos et al., using the capture and recapture technique with data from SINAN, SISCEL, and clinical records in Sergipe, Brazil, have estimated an underreporting of 34.3%^8^. Underreporting was lower in Vitória, from 2000 to 2006, reaching 4.9% for HIV-infected pregnant women^9^.

The identification of cases of HIV-infected pregnant women in other Health Information Systems, mainly in the SICLOM and SISCEL, followed the same pattern observed in the routine used by the Ministry of Health to identify cases of AIDS in adults and children. According to the last epidemiological bulletin of the Ministry of Health^1^, of the 39,113 registered cases of AIDS in 2015, 56.3% were from the SINAN, 7.4% from the SIM, and 36.3% from the SISCEL/SICLOM.

Evaluating the data from HIV-infected pregnant women and children in five states and the Federal District, Miranda et al. have shown that the best source to find children under 13 years of age with HIV was the SISCEL; SINAN contained less than a third of the cases^10^.

According to the results of this study, the search in the HIS would allow the increase in the notification of cases of HIV in pregnancy by 57.0% and the system that would result in the identification of most new cases would be the SICLOM. According to the Ministry of Health^1^, a new functionality in the SICLOM was developed to inform HIV/AIDS-infected patients who have not yet been reported in the SINAN. This functionality could be used for the cases of HIV in pregnancy, identifying women not reported during pregnancy in the provision of antiretroviral therapy.

One possibility not investigated in this study would be the search for pregnant women in the Hospital Information System (SIH) who were hospitalized for abortion. Although restricted to public services, the Hospital Information System would be the only one that would allow the identification of HIV-infected pregnant women whose outcome of pregnancy was an abortion. A study carried out in Rio de Janeiro, Brazil, which aimed to estimate the incidence of congenital syphilis in pregnant women in the SUS network^11^, has carried out the search for cases in the Hospital Information System, which resulted in the identification of two pregnant women infected with the disease who had not been reported in the SINAN. In addition to increasing the number of cases of congenital syphilis and syphilis during gestation, this search allowed the identification of a negative outcome of syphilis infection during pregnancy (abortion) that would have been lost by the health information systems. However, the data in this study do not allow us to estimate the type of outcome that would be obtained in the case of HIV infection.

In this study, the pregnancies in the period were identified in the database of the “*Nascer no Brasil*” study. The Live Birth Information System (SINASC) – a system with national coverage, covering births in public and private hospitals and households – would be the system to be used to identify pregnancies that resulted in live births. On the other hand, the SIM, also with national coverage although with regional variations, would be the system to be used for the identification of pregnancies that resulted in fetal deaths. Although the Hospital Information System only covers hospital admissions in the public sector, it could be used to identify pregnancies that resulted in an abortion.

All the cases of HIV-infected pregnant women identified in the Health Information Systems were located by the maternity hospitals that participated in the “*Nascer no Brasil*” study, as the undiagnosed cases occurred in a gestation after the one evaluated by the study or were not confirmed in the current gestation. This result suggests that mechanisms for the identification of HIV-infected pregnant women have been used in larger maternity hospitals, with more than 500 births per year, and the underreporting identified is due to failures in the filling or sending of reporting records. As the serological testing for HIV during prenatal care has not reached 100% coverage in Brazil, diagnostic mechanisms at admission for delivery, such as the use of rapid tests, have been used and they need to be constantly used to avoid missed opportunities for the diagnosis of HIV. Since the “*Nascer no Brasil*” study did not include maternity hospitals with less than 500 births per year, or births at home or on public roads, the missed opportunities for the diagnosis of HIV infection in these contexts should be investigated in future studies.

This study presents some limitations. The “*Nascer no Brasil*” study included only pregnant women admitted to hospitals with more than 500 births pear year and it excluded women hospitalized for abortion. Therefore, women identified as HIV-infected pregnant women in this study do not represent the universe of Brazilian pregnant women. We could not evaluate the coverage of the SINAN Pregnant Women in these cases. The study also did not perform serological tests for HIV, and the identification of cases was based on secondary data from prenatal and medical records. There are possible errors in the identification of pregnant women, especially if the women who performed only one rapid test at admission for delivery were classified as HIV infected, without further confirmation. Finally, we could not analyze the SIM database, which hindered the linkage with this health information system. However, the SIM, as well as the SISCEL and the SICLOM, are routinely related to the SINAN. The nominal search in the SISCEL and the SICLOM did not result in additional cases to those identified in the SINAN database. Thus, we believe that the lack of a linkage with the SIM database has not lead to a loss of cases.

This study has identified a significant underreporting of HIV-infected pregnant women in the SINAN. The search for cases in other Health Information Systems, a routine already adopted by the National STD, AIDS, and Viral Hepatitis Department for cases of AIDS in adults and children, would increase the notification of cases of HIV in pregnancy. This measure would be an addition to the incentive to increase the reporting of cases of HIV in pregnancy, since several data relevant to the surveillance of this disease are available only in the SINAN.
